# Deliberate self-harm: Case identification and incidence estimate upon data from national patient registry

**DOI:** 10.1371/journal.pone.0231885

**Published:** 2020-04-27

**Authors:** Ping Qin, Lars Mehlum

**Affiliations:** National Centre for Suicide Research and Prevention, Institute of Clinical Medicine, University of Oslo, Oslo, Norway; Fayoum University Faculty of Computers and Information, EGYPT

## Abstract

**Background:**

Patient registry is a valuable source for identification of deliberate self-harm (DSH) incidents on a population basis; however, little research has provided sufficient details that could inform best utilization of this complicated data source for DSH research and statistics. In this study we want to use data from the Norwegian Patient Register to delineate a diagnostic coding system to identify medically treated DSH incidents, to profile general characteristics of these incidents, and to estimate incidence rate of DSHs in the national population of Norway.

**Methods:**

We obtained all records of patient contacts to somatic hospitals and emergency centers due to external causes from 2008 through 2013. For each contact, we retrieved clinical data on primary and comorbid diagnoses, urgency of contact, etc., alongside with personal general information. After careful mining, the data were further processed following a multi-step analytic strategy. Descriptive analysis was used to profile DSH incidents and to estimate incidence rates and corresponding 95% confidence intervals.

**Findings:**

From 1 459 384 eligible incident contacts for emergency injury treatment, we identified 13 533 incidents that had a recorded diagnosis of DSH, i.e., with a supplemental code of X6n. Upon delineation of these recorded DSHs, we devised a diagnostic coding system to identify all possible DSH incidents. This yielded a total of 38 433 incidents to be considered as likely incidents of DSH (adjusted incidents)–a number that is 2.84 times higher than that of the recorded DSH incidents during the study period. While the proposed diagnostic system captured more incidents by males and old adults, the adjusted DSHs remained more common in females than males, and occurred most frequently within young people of 18–21 years. These incidents imply about 6400 DSH incidents from 5100 persons per year and correspond to a yearly cumulative incidence rate of 121.0 (95% CI: 113.4–128.2) per 100000 population over 10 years old with particularly high rates in teenage girls of 15–19 years old (296.1/100000) and females and males of 20–24 years old (282.5 and 178.8/100000, respectively).

**Conclusion:**

The insightful details of data processing and the rich findings from this study underscore the importance of patient registry in studying and reporting DSH incidents on a population level.

## Introduction

Deliberate self-harm (DSH) is an important health problem in many parts of the world; however, accurate identification of DSH incidents and report of its incidence rate on a national basis have been challenging tasks. Although many countries have a national vital registration system to record, collect and process information related to suicide mortality, few country has an equivalent system specifically dedicated to non-fatal suicidal behavior or deliberate self-harm [1]. The lack of systematic DSH registration leaves a large gap in our understanding of the non-fatal self-harming cases. It also hampers our efforts to accurately monitor DSH incidence, demographic patterns and temporal changes of self-harming methods in the population, and thus adversely affects development and evaluation of suicide prevention strategies at various levels.

To gain a picture of DSH incidence in a population, two primary methods have been used. The first is upon self-report of the behavior during a given time in surveys of representative samples of residents. This method may have the advantage of capturing untreated episodes of DSH, but is prone to common shortcomings of questionnaire surveys, such as low reliability and precision of data, selection and information biases, insufficient response rates, limited number of participants to be surveyed, etc. [1, 2]. Another method to obtain the data is through retrieval of data from medical records on DSH episodes treated in health services in the community. Although this method is criticized for only being able to include severe forms of DSH leading to medical attention [1, 3], it provides the very best opportunity to obtain reliable and precise data of DSH with the capacity to cover a large population.

Utilization of patient records to identify DSH incidents for a large or national population has become an increasing trend for research in recent years. For instance, there have been studies on DSH or suicide attempt using the Veteran Healthcare System Database in the USA [4], the multicenter self-harm database in the UK [5], the national patient registry in Sweden and Denmark [6–8], and the national self-harm registry in Ireland [9]. To our awareness, however, no study has provided sufficient details on how DSH incidents were identified in the complexity of medical records in patient registries related to clinical priorities and reporting practices.

In the present study, we want to use the rich but underutilized source of data in Norwegian Patient Registry, referred to as NPR herewith [10], to profile the records of documented DSH incidents, to delineate a data-derived diagnostic algorithm of coding system to be used for identifying all possible DSH incidents from patient records, to profile general characteristics of the DSH incidents and moreover to estimate the incidence rate of the DSH in the national population of Norway. We believe the approach and process we adopted in data handling as well as the outcome we produced from the study are informative for studying DSH with data from patient registries or administration databases.

## Materials and methods

### Data sources

Our primary data source, the Norwegian Patient Registry (NPR), covers the entire national population of Norway with data on a personal identifiable level since the year 2008 [10]. It contains data on both somatic and psychiatric contacts with specialist healthcare services, i.e., hospitals and associated emergency rooms, ambulance and outpatient clinics as well as contracted private specialists in Norway. For each contact or treatment it records date of contact, primary and secondary diagnoses, type of contact, urgency for treatment, place of discharge along with other details such as whether the contact was being planned or emergent, and service unit for treatment, etc. Diagnoses of illness are in accordance to the Norwegian version of the International Classification of Diseases, 10th edition (ICD-10) [11]. For contacts elicited by external causes, a supplementary classification, referred to as E-codes, could be used to further describe the nature of the condition, e.g., a supplement code ‘X6n’ refers to intentional self-harm. This register enables us to identify individuals who have been in contact with the specialist healthcare services for treatment due to self-inflicted injury, accidental harm, injury enforced by others, etc.

Other data sources include the Norwegian Central Population Registry and the Statistics Norway’s Events Database (the so-called FD-Trygd database) [12, 13]. The central population registry contains the unique personal identification and general demographic information such as sex and birthdate for all residents in the country. The Statistics Norway’s Events Database, available since 1992, contains personal running data on demographic and socioeconomic status. These registers allowed us to obtain personal information on sex, birthdate and place of residence along with the personal identification to be used for interlinking personal data across registries. Numbers of the national population, by sex and age on the 1^st^ of January each year during the study period, were derived upon the data published at the homepage of Statistic Norway [14].

### Procedure of data mining

**Fig 1:**
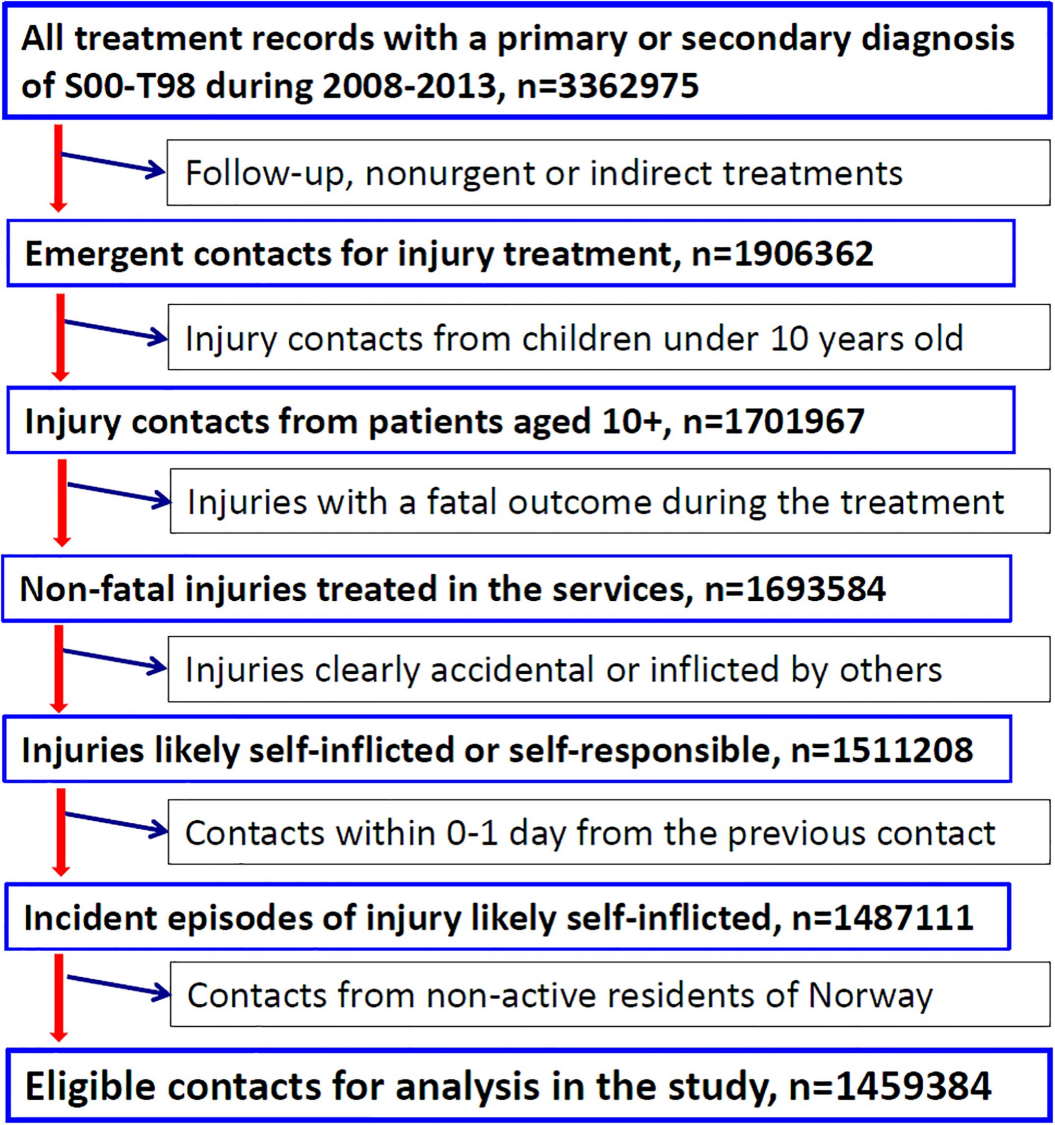
Flowchart of the process selecting eligible incidents of injuries treated in the secondary health care in Norway during 2008–2013.

From the NPR database, we obtained all episode contacts (n = 3 502 707) for treatment because of external causes, i.e., poisoning or injuries coded as S00-T98 in ICD-10 as the primary or a secondary diagnosis, from the year 2008 through 2013. For these contact records, we undertook a restricted process of data mining as shown in Fig 1. Firstly, we excluded types of contact being meeting discussion, follow-up treatment, rehabilitating and indirect patient contact, and thereafter the contacts that were not for emergency treatment. For the remaining contacts, we removed all contacts for children less than 10 years old because poisoning or injuries in children that young are unlikely to be deliberately self-inflicted. We then excluded episode records of poisoning or injuries that resulted in a fatal outcome, retaining only non-fatal incidents for our consideration. Moreover, we removed all records that were reported as accidents, war-related injuries, secondary medical injuries, injuries inflicted by other people or induced by environmental causes, i.e., having a code of V0-X5, X8n, Y3n, Y4n-Y86 or Y88-Y98 in the primary or secondary diagnoses and without a comorbid code of X6n, X6 or Y87. Thereafter, we excluded episode records that were reported 0–1 day from the previous episode because they were likely duplicate reports of the same incident made by treatment involved staff from multiple clinical departments or transferals from one department to another. For a few instances where an episode was reported more than once on the same date, the one with a comorbid diagnosis of deliberate self-harm (coded with X6n, X6 or Y87) received a priority to remain in the database for study; otherwise the first record sorted alphabetically by the primary diagnosis was retained. Lastly, we excluded records of treatments of people who, at the time of receiving the treatment, did not have a permanent residence address in Norway, keeping only records of contact from active residents in the country.

After having gone through the procedures described above, a total of 1 459 384 contact records were finally considered as eligible incidents, i.e., non-fatal injuries or poisonings from residents of 10 years and above that were likely self-inflicted or self-responsible and that received urgent direct medical treatment in the specialist healthcare services in Norway. These incidents formed the basis of incident contacts for identification of DSH incidents in the present study.

### Definition of variables

Based upon the details of data derived from the NPR we constructed a range of variables to be used in the analyses. Clinical diagnoses of our study interest were defined and categorized in concordance with the Norwegian version of the ICD-10 [11].

1. Recorded deliberate self-harm: A recorded DSH was defined by having a code of X6n or Y87 in the primary or secondary diagnoses in connection to the poisoning or injury being treated. This supplemental diagnosis is reported by first-line clinicians, usually upon the information from patients themselves or accompanying persons at the time of arrival for medical treatment. The codes of DSH are often underreported and are normally given as a secondary diagnosis with rare exceptions.
2. Form of injuries: The form of injuries was grouped into 6 categories, including 1) poisoning with medications (G1), 2) poisoning with other substances (G2), 3) injury on forearms, hands, wrists and arms (G3), 4) injury on other specific parts of body (G4), 5) injuries by suffocation, embedment of foreign objects, drowning and burning, etc. (G5), 6) other injuries not covered above (G6). Exact codes in ICD to be included in each category are shown in the panel (Table 1) and were delineated upon the data analyses examining diagnostic codes of injury most relevant to DSH.
3. Psychiatric comorbidity: Comorbid diagnoses of psychiatric illness were defined by the ICD codes of F0-F9 in the primary and secondary diagnoses. For each injury contact, we extracted data on presence of psychiatric diagnoses and analyzed the diagnosis in the context of injury form.

**Table 1:** Distribution of the recorded deliberate self-harm incidents and the adjusted incidents of deliberate self-harm identified by the proposed list of diagnostic codes from the Norwegian Patient Registry.

Cause of contact	Diagnostic codes in ICD-10^†^	DSH incidents recorded with X6n^‡^		DSH incidents identified by the proposed coding system		
		Distribution by primary diagnosis n (%)	With co-morbid diagnosis of F0-F9 n (%)	Distribution by primary diagnosis n (%)	With co-morbid diagnosis of F0-F9 n (%)^‡^	With co-morbid diagnosis of X6n n (%)^‡^
**G1**: Poisoning with medication and biological substance	T4n, T50, T96	9912 (73.2)	5646 (57.0)	27639 (71.9)	12787 (46.3)	9912 (35.9)
**G2**: Poisoning with other substance	T51-T55, T57-T60, T62,T65	346 (2.6)	161 (46.5)	1460 (3.8)	1275 (87.3)	346 (23.7)
**G3**: Injury on hand, forearm, overarm/shoulder	S41, S45, S50, S51, S54-S56, S59, S61, S64-S65, S69	450 (3.3)	147 (32.7)	699 (1.8)	396 (56.7)	450 (64.4)
**G4**: Injury on other specific body parts	S10, S11, S15, S17, S19, S21, S25-S27, S31, S35-S39, S71, S88, T01, T09, T11	418 (3.1)	251 (60.1)	834 (2.2)	667 (80.0)	418 (50.1)
**G5**: Suffocation, embedment of foreign objects, burning, etc.	T18, T19, T27, T28, T31, T68, T69, T71, T95	281 (2.1)	175 (62.3)	862 (2.2)	756 (87.7)	281 (32.6)
**G6**: Other injuries	Codes in S0-T9 not listed above	582 (4.3)	225 (38.7)	974 (2.5)	801 (74.8)	582 (59.8)
**G7**: Self-harm, intentional	X6n: X6, Y87	24 (0.2)	9 (37.5)	24 (0.1)	9 (37.5)	24 (100.0)
**G8**: Physical illness	*	587 (4.3)	336 (57.2)	2198 (5.7)	1947 (88.6)	587 (26.7)
**G9**: Psychiatric disorder	F0-F9	933 (6.9)	933 (100.0)	3743 (9.7)	3743 (100.0)	933 (24.9)
**Total**		**13533 (100.0)**	**7883 (58.3)**	**38 433 (100.0)**	**22197 (57.8)**	**13533 (35.2)**

^**†**^: Codes listed in G1-G6 were restricted to diagnostic codes under which a recorded DSH was present in more than 1% of their incident contacts and the few codes (S50, S59, S61, S69 and T01) of which a recorded DSH was present in many incident contacts.

^‡^: Data were based on both primary and secondary diagnoses.

*: all codes about somatic illness.

### Statistical analysis

Descriptive analysis was used to profile deliberate self-harm incidents from eligible contacts recorded in the source database. For the purposes of the present study, we undertook the following multi-step strategy to analyze the data step by step. Firstly, we examined all eligible contacts with a recorded diagnosis of DSH to examine the compositional distribution of injuries or medical conditions that were often reported with this comorbid diagnosis. If a comorbid diagnosis of DSH was present in at least 1% of a primary diagnosis in all eligible contacts, this contact was then grouped, by the ICD code of its primary diagnosis, into a corresponding category by form of harm or else category as shown in the panel (Table 1). We also included a few diagnostic codes (ICD codes: S50, S59, S61, S69 and T01) that are clinically common in DSHs albeit a recorded DSH was present in less than 1% of these incidents. Secondly, upon the findings from the first step and our current knowledge about DSH we devised a data-driven diagnostic algorithm of coding system to be used to identify incidents that were likely DSHs from the eligible incident records, with priorities given to contacts with a recorded diagnosis of DSH, psychiatric comorbidity and form of harm or injury hierarchically. Thirdly, we profiled the general and clinical characteristics of the adjusted DSH incidents identified by the proposed coding system, and compared that with the recorded DSH incidents via Chi-Square test. As the final step, we estimated the yearly incidence rates of DSH and their 95% confidence intervals in the national population of Norway during the study period. All analyses were carried out using SAS/STAT software, version 9.4 of the SAS System for Windows. Copyright © [2019] SAS Institute Inc., Cary, NC, USA.

### Ethical consideration

The study was approved by the South-East Regional Ethical Committee for Medical Research (2013/1620/REK Sør-Øst) and by the owners of the registries from which the data were used in the present study. As the study was conducted with the use of register-based data and without any patient contact, the ethics committee waived the requirement for informed consent. Patient information was pseudo-anonymized prior to data analysis and stored under existing legislation. All data presented in public are fully anonymized ensuring that no single participant or group is recognizable.

## Results

### Examination of diagnostic codes in recorded DSH incidents

From 1 459 384 eligible incident contacts for emergency injury treatment during the 6-year period of study, we identified 13 533 incidents (0.93%) that had a recorded diagnosis of DSH, comprising 4 175 contacts from men and 9 358 from women. Table 1 shows the distribution of the primary diagnosis of these recorded DSH incidents, by specific form of injury or harm with ICD codes that a diagnosis of DSH was frequently reported along with.

Poisoning with medications (G1) was the most frequent primary cause for the contacts with a recorded DSH, accounting for 73.2% of these incidents. Injuries on forearms, hands, arms and other specific body parts (G3-G4) as well as in forms of suffocation, drowning, embedment of foreign objects and burning (G5) together were the primary diagnosis for 8.5% of these incidents. Although an acute harm or injury was, as an inclusion criterion, involved in all of these incidents, physical illness (G8) was the primary diagnosis in 4.3% and psychiatric disorder (G9) in 6.9% of these incidents.

At the same time, a comorbid diagnosis of psychiatric disorder was present in 58.3% of all incidents with a recorded DSH, indicating the importance of psychiatric comorbidity in reporting an injury or poisoning as a DSH by first-line clinicians.

### Identification of adjusted DSH incidents

Based upon the results from examining incidents with a given diagnosis of DSH and our current knowledge of DSH as well as experience from countries such as Denmark [15], we devised a data-driven diagnostic algorithm of coding system to identify all possible DSH incidents from the eligible contacts. We undertook a 4-step hierarchical consideration as described below: 1) all incidents with a recorded DSH in either primary and secondary diagnoses (13 533 incidents included), 2) incidents with a primary diagnosis listed in G1-G5 and a secondary diagnosis of psychiatric disorder (9 501 incidents included), 3) incidents with a secondary diagnosis of injuries listed in G1-G5 and a primary or secondary diagnosis of psychiatric disorder (4 813 incidents included), and 4) incidents with a primary or secondary diagnosis of medicament poisoning (G1) not covered in the above steps (10 586 incidents included). In total, 38 433 incidents were considered as most likely incidents of DSH, i.e., the adjusted DSH incidents, that received a medical treatment in hospitals and associated emergency services during the 6-year period from 2008 to 2013 –a number that is 2.84 times higher than that of the recorded DSH incidents.

Table 1 also shows the distribution of the adjusted DSH incidents by the primary diagnosis, the presence of a recorded DSH and psychiatric comorbidity. Proportionally, the adjusted DSH incidents distributed consistently with the recorded DSH incidents in their primary diagnosis with poisoning being the majority, but there are also notable differences (χ^2^ = 544.7, p<0.001). The adjusted DSH incidents include slightly less of injuries (G3-G5), especially those listed in G3, but relatively more of psychiatric illness (G9) being the primary diagnosis. Poisonings (G1 and G2) accounted for 75.7% of the adjusted DSHs, including 71.9% with medications and 3.8% with other substances, whilst physical illness (G8) was the primary diagnosis in 5.7% of the adjusted DSH incidents.

At the same time, a comorbid diagnosis of psychiatric illness was present in 57.8% of the adjusted incidents of DSH, with a relatively low presentation in poisonings with medications (G1, 46.3%) and injuries on forearm, hands and arms (G3, 56.7%) and a high presentation in injuries on other body parts (G4, 80.0%), injuries by methods of suffocation, embedment, burning, drowning and jumping (G5, 87.7%) and poisonings by other substances (G2, 87.3%). A recorded X6n was present in 35.2% of the adjusted incidents of DSH. This supplemental diagnosis was more often reported for injuries on forearms, hands and arms (G3: 64.4%) but least often in poisonings with other substances (G2, 23.7%) and incidents for which physical or psychiatric illness was the primary diagnosis. Overall, the presence of psychiatric comorbidity in the adjusted DSH incidents was comparably common as that in the recorded DSH incidents (57.8% vs 58.3%, χ^2^ = 2.09, p = 0.15).

### Characteristics of DSH incidents identified through the proposed coding system

According to the proposed diagnostic system, we identified approximately 6400 incidents of DSH that were treated at the specialist healthcare services each year, with small fluctuations across calendar years during the study period (Table 2). Clearly, the adjusted DSH incidents captured some more incidents in the early years when the supplement diagnosis of DSHs was relatively less frequently reported (χ^2^ = 39.3, p<0.001).

**Table 2:** General characteristics of the 13533 incidents with a recorded DSH and the 38433 incidents of DSH identified by the proposed coding system.

Variable	Recorded DSHs N = 13533		Adjusted DSHs N = 38433		Test of difference
	N	%	N	%	
**Calendar year**					χ^2^ = 39.3, p<0.001
2008	2183	16.1	6479	16.9	
2009	2109	15.6	6352	16.5	
2010	2196	16.2	6193	16.1	
2011	2261	16.7	6431	16.7	
2012	2325	17.2	6489	16.9	
2013	2459	18.2	6489	16.9	
**Sex**					χ^2^ = 646.7, p<0.001
Female	9358	69.1	23271	60.5	
Male	4175	30.9	15162	39.5	
**Age group**					χ^2^ = 998.2, p<0.001
10–19 years old	2305	17.0	5277	13.7	
20–34 years old	5145	38.0	13405	34.9	
35–49 years old	3527	26.1	9799	25.5	
50–64 years old	1877	13.9	5777	15.0	
65–79 years old	520	3.8	2277	5.9	
80+ years old	159	1.2	1898	4.9	
Type of harm involved*					
Poisoning	11698	86.4	34152	88.9	χ^2^ = 123.6, p<0.001
With medication (T4n-T50, T96)	11310	83.6	32026	83.3	
With other substance (T51-T65, T97)	682	5.0	2867	7.5	
Injury of any body part	2182	16.1	5264	13.7	χ^2^ = 104.1, p<0.001
On head (S00-S09, T90)	281	2.1	532	1.4	
On hand, forearm, arm and shoulder (S40-S69, T91, T92)	745	5.5	1519	4.0	
On other body parts (S10-S39, S70- T14, T93, T94)	867	6.4	1932	5.0	
Suffocation, embedment, drowning, burning, etc. (T15-T35, T66-T78, T95)	474	3.5	1712	4.5	
Injury or poisoning involving diagnostic codes listed in G1-G5	13001	96.1	37901	98.6	χ^2^ = 992.6, p<0.001

*: Both primary and co-morbid diagnoses were considered when examining form of injuries involved, therefore the distribution across categories is not mutually exclusive.

While both the recorded and adjusted DSHs were more common in females than males, 39.5% of the adjusted DSHs were incidents by males, which is a significantly larger proportion than that in the recorded DSHs (30.9%) (χ^2^ = 646.7, p<0.01). At the same time, the adjusted DSHs included relatively more incidents from adults of high ages (χ^2^ = 646.7, p<0.01). For instance, DSHs of people over 64 years old accounted for only 5.0% of the recorded DSHs, but this proportion increased to 10.8% in the adjusted DSHs. Still, a goodly amount of the adjusted DSHs were from adolescents and young adults.

**Fig 2:**
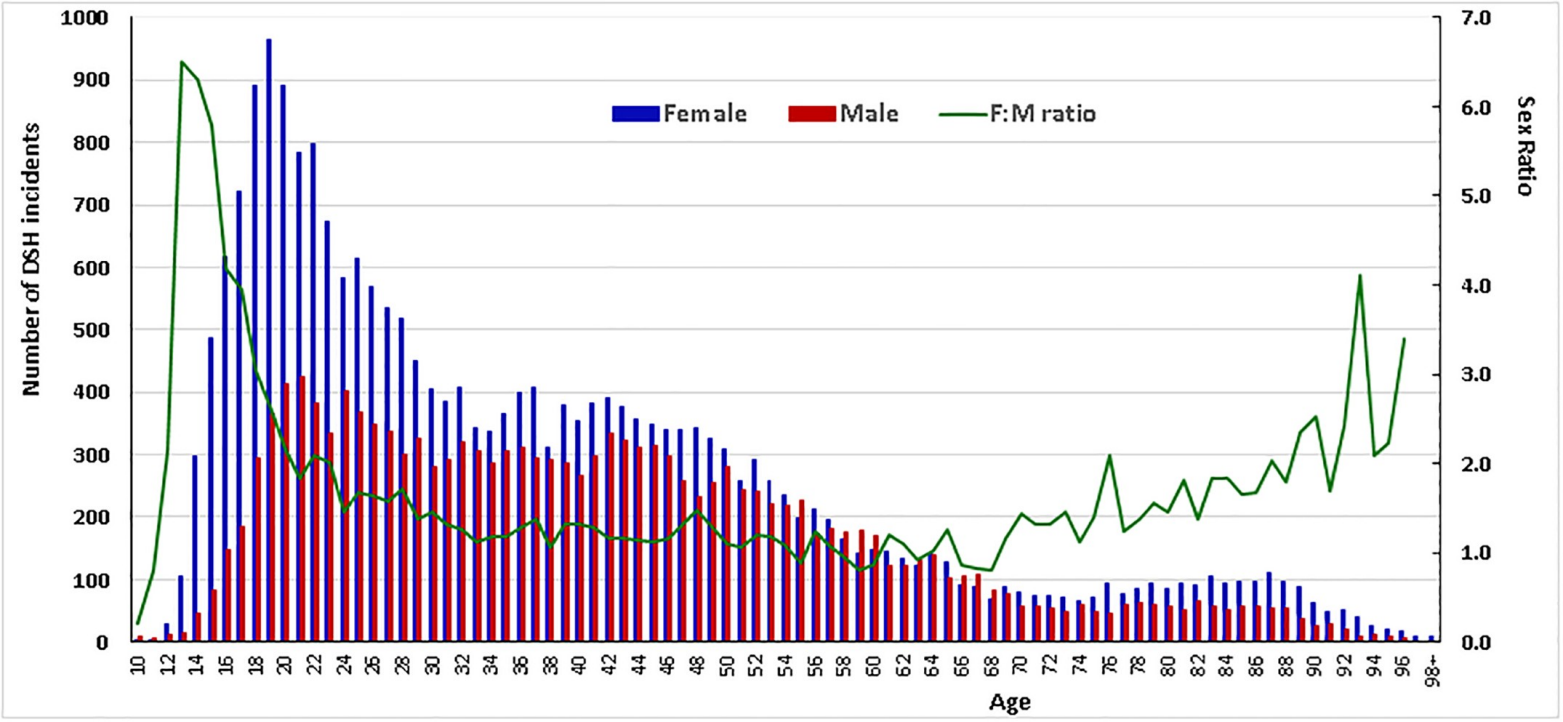
Sex and age distribution of DSH incidents identified by the proposed diagnostic codes from the national patient registry from 2008 through 2013.

On average, we identified around 3880 female and 2530 male incidents of possible DSH being treated in specialist healthcare services each year. This corresponded to a ratio of female to male of 1.53 during the study period, but the sex ratio varied substantially by age. As shown in Fig 2, the adjusted incidents of DSH increased dramatically from early adolescence to young adulthood and peaked at the ages of 19–20 years old. The observed age-specific phenomenon was particularly striking among young girls, with a vast increase of DSHs in adolescent girls of 13–16 years old and a sex ratio of female to male over 4 to 6 times. While the adjusted DSHs remained more frequent in females than males of most age groups, a reversed sex ratio was noticeable among adults of 55–67 years old.

Table 2 also displays the distribution of harm or injury forms involved in the adjusted DSH incidents as being coded in either primary or secondary diagnoses. 88.9% of the adjusted incidents involved overdose of medication (83.3%) and/or poisoning with other substances (7.5%). Body injuries were involved in about 13.7% of the adjusted DSHs and specifically, wounds on hand, forearm and arm were present in 4.0% of these incidents. Clearly, the adjusted DSHs captured relatively more incidents involving poisonings, especially with substances other than medication, but less incidents involving injuries as compared with the recorded DSHs. Overall, 98.6% of the adjusted DSH incidents involved a form of injuries or harm for which the diagnostic codes were listed in the groups G1-G5—a significant larger proportion than the 96.1% of the recorded DSH incidents (χ2 = 992.6, p<0.001).

### Cumulative incidence (incidence rate) of DSH in the national population

According to the proposed diagnostic system, about 5130 individuals each year (2930 females and 2200 males) received emergent somatic treatment because of DSH during the study period. This corresponds to a yearly cumulative incidence rate of 121.0 (95% CI: 113.4–128.2) per 100 000 population over 10 years old in Norway, and a rate of 133.4/100 000 (95% CI: 121.7–145.1/100 000) for females and 108.6/100 000 (100.6–116.6/100 000) for males above 10 years old.

**Fig 3:**
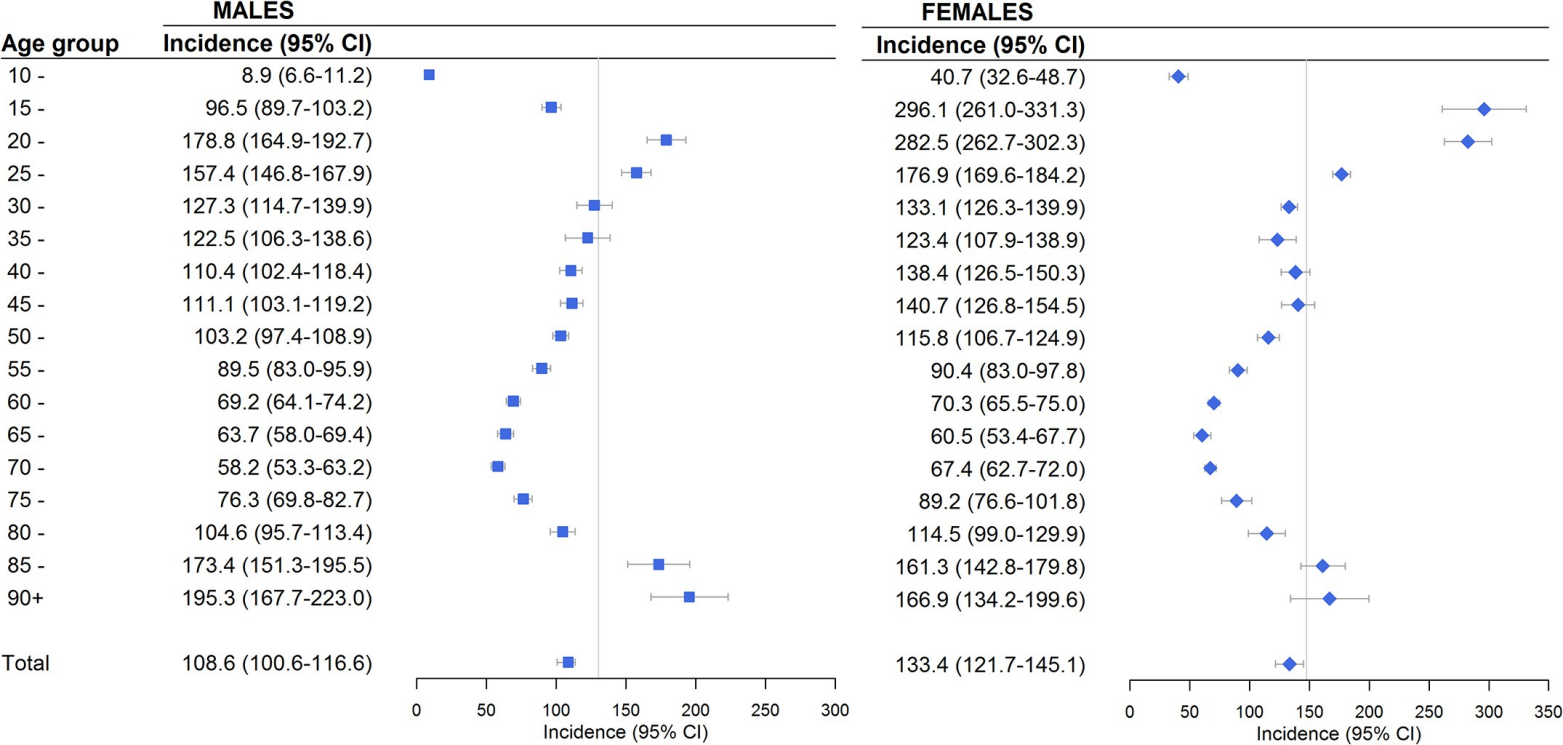
Estimated incidence rates of deliberate self-harm by sex and age groups in the population during 2008–2013 (per 100 000 population).

As shown in Fig 3, the incidence rate of DSH varied greatly by age in both sexes with two clear peaks—one among the young and another among the old elderly. For the young, the cumulative incidence rate was highest for teenage girls of 15–19 years (296.1/100 000) and young females and males of 20–24 years old (282.5/100 000 and 178.8/100 000, respectively). While the incidence rate of DSH was relative low among adults in their 30s to 50s, it was the lowest in people of 65–74 years old, and then increased sharply with increasing age among the elderly of both sexes.

## Discussion

### Validity of the proposed diagnostic system to identify DSH incidents

Patient records on medical treatment accumulated in contemporary health care systems provide a valuable source for studying deliberate self-harm, for which research validating the records to best identify DSH incident cases is a prerequisite. In the present study we were unable to adopt typical methods for validation of medical diagnoses, i.e., via additional information from external sources to provide a measurable estimate of validity of our proposed diagnostic system for DSH identification (to produce Positive Predictive Value, Specificity and Sensitivity) [15]. Instead, we analyzed characteristics in the patient records with a valid diagnosis of DSH and, in combination with existing knowledge on DSH, developed a data-derived diagnostic algorithm of characteristics that could be used to identify likely cases of DSH in the Norwegian Patient Registry. In recent years, utilization of developing internal diagnostic algorithms to identify cases has become increasingly common in medical research [16]. This method is quick and incurs no extra cost, and has the potential to validate diseases for which specific treatment routines are used universally in the catchment area. To our awareness, however, few studies so far have used it to validate specific diagnostic codes. The use of such algorithms upon patient data may exclude less severe cases, such as self-harm in mild forms that do not lead to medical treatment. On the other hand, robust validations requesting additional information are limited in size due to costs, and their generalizability is compromised by nonparticipation of many practices. Careful use of internal diagnostic algorithms may overcome these limitations, and represent a cost-effective method of identifying valid individual cases.

In order to persevere with the definition of DSH incidents, we applied a comprehensive procedure for data mining, ensuring that only actual new incidents of harm and injury were retained as eligible for further consideration. For instance, a diagnosis of injury, in form coded within S00-T98, was an essential requirement for selecting eligible incidents for consideration. Such a restriction would exclude most patient contacts involving only serious suicidal ideation. At the same time, the multi-step procedures we took for data mining, as described in Fig 1, eliminated possible duplicative reports of same injury incident, injuries that were clearly accidental or not new incidents, and injuries by small children or non-active residents as well as injuries with fatal outcomes. These procedures are crucial in studies of DSH incidence when utilizing administrative databases of patient records collected accumulatively. The present study is, to our awareness, the first that has provided a clear and thorough description of the processes of data mining to select eligible records for further consideration.

Certainly, the accuracy of data on either recorded or adjusted DSHs relies on the quality of registration work performed by clinicians. In emergency clinics, assessment of intentionality for injuries is often not among the priorities during treatment. As shown in our data, only 13533 injury incidents, around 2250 per year, were reported as DSH, i.e., with a supplemental diagnosis of X6n, X6 or Y87, which implies a clear underreporting of DSH in the patient registry. This was especially evident in early years of the study period and in contacts from patients of high ages. Compared with the recorded DSHs, the proposed diagnostic system captured relatively more DSH incidents in the early period of study and incidents from males and people of higher ages. Overall, the incidents of DSH identified by this diagnostic system almost tripled the number of recorded DSH incidents to approximately 6400 per year—a number that is highly comparable to the often quoted estimate in Norway, although we could not relocate any scientific source for this estimate.

While increasing research on DSH have used data from patient registries, there is high variability between studies in their methods used for identifying DSH incidents from the source databases. For instance, to ascertain incidents being DSH, the Irish national self-harm registry had the data examined by data registration officers following the Registry’s standardized methods of case ascertainment [9]; the UK multicenter self-harm study established a panel of experts to diagnose DSH/SH [17]; Studies from Sweden normally used the recorded ICD codes for DSH (E950-9, X60-84) and uncertain causes (E980-9, Y01-34) to identify DSH cases from the Swedish national patient registry [7]. Denmark performed a validation study with external data to produce a diagnostic system to be used to identify possible DSH incidents from the national patient registry [15]. This recommended diagnostic system has been adopted in a number of studies on DSH from Denmark [6, 18, 19] and is highly comparable to our devised diagnostic system.

Despite the rationale and the basis of our devised diagnostic system for DSH incident identification being data-driven, our adjusted DSH incidents may not be the true number, but one step closer. There is a clear need to find methodological systems for ascertaining data on DSH incidents on the national and regional levels that are both practically viable and scientifically valid. It is also desirable to have further studies to validate the proposed algorithm and the results present in this study. We are currently planning one such validation study through utilizing data from other sources in Norway.

### Adjusted incidents of DSH and estimated incidence rate in Norway

A total of 38 433 DSH incidents, about 6400 per year, were identified by the proposed diagnostic system during the 6-year study period. Overall, 60.5% of these incidents were by females, 48.6% were by people under 35 years old, 57.8% occurred with a comorbid psychiatric diagnosis, and 88.9% involved poisoning with medication or other substances whilst 13.7% involved body injuries. The estimated annual incidence rate was 121.0 (95% CI: 113.4–128.2), 133.4 (95% CI: 121.7–145.1) and 108.6 (95% CI: 100.6–116.6) per 100 000 population, respectively, for the total, females and males over 10 years old in Norway.

Deliberate self-harm that receives medical treatment in specialist healthcare services represents DSH incidents of relative high severity with poisoning by medication being most common. Our finding of a 83.3% of the adjusted DSHs involving medication poisoning is consistent with the reports upon national data from, e.g., Sweden (83.8% of all DSHs recorded with E950-9, E980-9, X60-84, or Y01-34) [7], Ireland (drug overdose was the sole method used in 68% of all cases, 75% of female and 59% of male cases) [9] and Denmark [6].

Our observations of a very high incidence of DSH in adolescent girls and a female to male ratio of 4–6 among adolescents of 13–16 years old are in high concordance with previous studies reporting that self-harm was about five times greater in females than males of 12–15 years old [20]. This gender imbalance is substantially larger than the one reported in community surveys in most countries [21], reflecting that a relatively larger proportion of self-harming females than males receive treatment in secondary health care. The number of comparable studies of hospital admitted DSH in the elderly is limited, but the level of DSH in the elderly in our study was considerably higher than previously published rates [6].

The high presentation of psychiatric comorbidity in DSHs has been well documented in both clinical and population studies [22]. Our data indicates that as high as 57.8% of the adjusted DSH incidents occurred with a psychiatric comorbidity when the patients were presenting to hospital services for medical treatment. While this is somewhat lower than the comorbidity of over 80% reported in a systematic review that included only studies in which an assessment of psychiatric comorbidity was made when self-harming patients presented to hospitals [22]; it is highly in line with the studies with population data of DSHs derived from patient registries [7, 9, 15].

Our estimated annual incidence rates of 108.6/100 000 for men and 133.4/100 000 for women are somewhat comparable to the incidence rates in Denmark (86.9/100 000 for men and 130.7/100 000 for women) that were also estimated upon data derived by the recommended diagnostic system [6]. These rates, however, are notably lower than the reported incidence rates in Ireland (Annual rate for the total, males and females was 198 (95% CI: 196–200), 173 (95% CI: 171–175) and 224 (95% CI: 221–226) per 100,000, respectively) [9]. It is difficult for us to tell whether this difference was caused by the substantially different methodological approaches used in the two studies or it represents real differences in DSH incidence rates between the two populations; Nevertheless, the data from Ireland seemingly cover relatively more incidents of self-harm by injuries [9].

### Limitation, strength and implication

The results of this study should be interpreted in the context of its limitations and strengths. It is evident that a large proportion of self-harm incidents occurs in the community and stays in the community without becoming known to clinical services [23]. Results from studies based only on hospital contacts should, thus, not be generalized to the larger group of self-harmers who do not need or seek hospital treatment for their injuries. Although Norway has a favorable health care system and almost all self-harm cases that become known to other people are referred for medical treatment, the DSH incidents identified from the NPR is still an underestimate of the national DSHs of various forms and severities in the country. The underestimation may also be caused by failures in the recording processes in the hospital settings. Despite our efforts in the data mining and delineation process, there were certainly incidents of injuries that were likely DSHs but lacking additional details such as comorbid diagnoses of X6n or psychiatric disorders and thus were uncaptured by our proposed algorithms for DSH identification.

Nevertheless, the present study is, to our knowledge, the first one to devise a data-driven diagnostic algorithm of characteristics that could be used to identify valid DSH incidents from a patient administrative database or registry. In lack of population level data on DSH incidents in Norway and internationally, application of the methodology adopted in this study could greatly enhance the utilization of patient data and intensify research on this topic in a timely manner. It makes use of patient registries, such as the NPR, as the valuable source of data for research on DSHs on a national level, and brings into light insightful details on how DSHs are recorded in the NPR database and how possible incidents could be identified with care.

DSH is a frequent cause of presentation to emergency clinics and results in a significant social and economic burden for communities because of the need of health services to treat the injury, the psychological and social impact of the behavior on the individual and families, and, occasionally, the long-term disability due to the injury. While we lack an established system continuously monitoring DSHs in the population, medical records from emergency and hospital services no doubt constitute a valuable source for monitoring on a population basis and for researching evidence to guide prevention strategies. The heavy underreport of DSH in patient records, as seen in the NPR, indicates that standardization of the reporting processes within countries, and subsequently between countries, is one of the basic tasks needed in our efforts to understand and eventually reduce DSHs. At the same time, our proposed diagnostic system, albeit subject to further validation with external information, provides a ground to start the utilization of this comprehensive and rich data source of the Norwegian Patient Register to search for epidemiological and clinical insights of medically treated DSHs, so that to inform acute somatic treatment and subsequent follow-up care of the patients with DSH.
